# Functional data analysis of FSH and inhibin B levels in four ovulatory cycles shows a changing oscillatory interaction during the follicular phase

**DOI:** 10.3389/fendo.2026.1741869

**Published:** 2026-05-14

**Authors:** Stephen J. Usala, A. Alexandre Trindade, David D. Vineyard

**Affiliations:** 1 Department of Internal Medicine, Texas Tech University Health Sciences Center, Amarillo, TX, United States; 2 Department of Mathematics and Statistics, Texas Tech University, Lubbock, TX, United States; 3 Department of Obstetrics and Gynecology, UT Southwestern Medical Center, Dallas, TX, United States

**Keywords:** dominant follicle, fertility, follicular phase, FSH, inhibin B, inhibins, oscillation, ovulatory cycle

## Abstract

**Objective:**

The pituitary hormone FSH (follicle-stimulating hormone) and the follicular granulosa cell hormone inhibin B are important in follicle development ending in ovulation. We hypothesized that FSH and inhibin B interact by oscillatory feedback during at least part of the follicular phase.

**Methods:**

Daily serum FSH and inhibin B were measured throughout the ovulatory cycles of four women. Transvaginal sonography was performed to establish the last day of the dominant follicle (DF) and the following day of its collapse. Serum FSH and inhibin B levels were indexed to day 0, the day of the ovulatory inhibin B peak, which occurred on the day of DF collapse in three cycles and the last day of the DF in one cycle. The out-of-phase day-specific serum FSH and inhibin B levels from the cycles were aligned with functional data analysis (fda) to generate standardized mean day-specific levels for meaningful comparison of FSH and inhibin B as a function of time-of-cycle.

**Results:**

The curves for the standardized mean day-specific FSH and inhibin B levels were related. A maximum positive correlation between FSH and inhibin B was achieved when the FSH curve was translated forward by 1 day relative to the inhibin B curve (curves were then “in-phase”). Similarly, translating the inhibin B curve forward by 1 day relative to the FSH curve resulted in a maximum negative correlation (curves were then “out-of-phase”). An oscillatory positive–negative feedback was discernible between FSH and inhibin B during the early follicular phase which appeared to change after day −8, the approximate timing for DF selection.

**Conclusions:**

Examining the relationship between inhibin B and FSH by fda using individual cycles has the potential to remove the obscuration that occurs from averaging multiple cycles. This exploratory study with fda revealed a possible FSH and inhibin B oscillatory feedback, **“**FSH<->B” (“FSH upregulates inhibin B, inhibin B downregulates FSH”), during the early follicular phase that qualitatively changes as the cycle progresses. We hypothesize that an oscillatory mechanism may have a role in the selection of a DF from a wave of developing follicles.

## 1 Introduction

The hormonal mechanisms that control ovarian antral follicular wave dynamics and selection of a dominant follicle (DF) during a menstrual cycle have not been fully elucidated. The progressive development of certain primordial follicles is considered the resultant of endocrine, paracrine, and autocrine factors that control follicular cell proliferation, growth, differentiation, and demise (1, 2). Pre-antral follicles (~0.1–0.2 mm) develop independently of luteinizing hormone (LH) and follicle stimulating hormone (FSH). At a diameter of ~0.2–0.4 mm, a fluid-filled cavity (called an antrum) begins to form around granulosa cells of the follicle; a cohort of follicles responsive to FSH grows in size that can be visualized by ultrasonography during an ovulatory cycle. This cohort or wave progresses and leads to the selection of a “dominant follicle” (DF), a follicle of ~10-mm diameter, that grows independently from FSH, unlike the subordinate follicles of the cohort which become atretic (1–5).

The present model for DF selection asserts that there is an FSH threshold above which FSH levels must be maintained in order to stimulate growth and development of a wave of antral follicles to a mid-point of the follicular (preovulatory) phase (2, 3, 6). After the start of menses, the days to reach this approximate mid-point can be variable. At the time point where the DF has reached suitable maturation in this follicular phase interval, the FSH level falls below a threshold, but the DF continues to grow in size and undergoes hormonal changes for approximately 7 days until ovulation. Put simply, a DF is established approximately 7 days before ovulation in the context of diminishing FSH levels. The uniqueness of the DF and its role in influencing levels of pituitary gonadotropins, the length of the follicular phase, and the timing of ovulation was shown in experiments where the DF was excised (i.e., follicle-ectomy) at various times prior to ovulation (7, 8).

Key negative regulators of the pituitary secretion of FSH are the ovarian hormones, the inhibins—inhibin A and inhibin B (3, 9–12). Inhibin A and inhibin B are members of the transforming growth factor-β superfamily and as ovarian hormones play autocrine, paracrine, and endocrine regulatory roles (10, 12). Inhibin A and inhibin B are each formed by a common α-subunit which is linked by a disulfide bridge to one of two highly homologous β-subunits, β_A_ and β_B_, resulting in α−β_A_ (inhibin A) and α−β_B_ (inhibin B), respectively (13–16). Both inhibin A and inhibin B can suppress FSH levels: FSH is upregulated in inhibin A knockout mice and suppressed in transgenic mice with upregulated inhibin A (17). FSH is the central regulator of ovarian follicular development, and activins are dimers of β_A_ and β_B_ which enhance FSH transcription and pituitary secretion (12, 18, 19). The present paradigm is that inhibins are competitive antagonists to activin action at the level of their corresponding pituitary receptors, which thereby reduce FSH transcription (9, 12).

Multiple studies have published the mean day-specific serum inhibin B and inhibin A levels indexed to the luteinizing hormone (LH) peak during the ovulatory cycle; in these studies, the presumptive day of ovulation, day 0, would be the day after the LH peak (20–25). The mean day-specific inhibin B profile is characterized by a) an increase starting the first day of menses and peaking ~day −9 to day −7, b) a nadir at day −2, c) a second peak at day 0, and d) post-ovulatory low levels with a rise before the start of the next menses. In contrast, the mean day-specific serum inhibin A profile is characterized by a) low levels in the early follicular phase, b) a rise beginning at the time of DF selection (~day −7), and c) high levels throughout the early and mid-postovulatory phases. Furthermore, inhibin B and inhibin A concentrations in follicular fluid of the developing follicle track the preovulatory serum levels (3, 25–29). Follicular fluid inhibin B concentration increases during the early follicular phase until a peak concentration at follicular diameter of ~9–11 mm, the size of a selected DF, and then sharply declines with larger follicular diameters. In contrast, the follicular fluid inhibin A concentration is relatively low until it starts to rise at ~9–10 mm. In summary, the hormonal time series for the follicular phase is as follows: max FSH -> max inhibin B/low FSH ~ DF appearance -> inhibin A rise (3, 30, 31). This time sequence suggests that inhibin B is the primary negative regulator of FSH in the early follicular phase until DF selection at the mid-follicular phase.

Not only does inhibin B suppress FSH levels in the first interval of the follicular phase, but also FSH increases inhibin B levels. FSH injections at 100-IU-stimulated inhibin B levels 36 h after treatment (32). Multiple studies selectively altering FSH levels during various intervals of the follicular phase, which was enabled by GnRH-deficient women as subjects and a GnRH antagonist in normal women, demonstrated that inhibin B levels are upregulated by FSH levels and that this control occurs in the early follicular to mid-follicular phases (33–35). FSH control of inhibin B occurs before the rise in inhibin A which begins after the time of DF selection. Furthermore, inhibin B levels during the early follicular phase are the resultant of FSH-modulated inhibin B secretion from smaller follicles at differing stages of development (3, 35).

The above results suggest that some form of an FSH, inhibin B positive-negative feedback mechanism might exist during the follicular phase of the ovulatory cycle. A positive–negative regulatory interaction, “FSH<->B,” suggested by the above introduces an important concept: the oscillatory dynamics of hormonal systems (36). The time function of pituitary–target tissue hormone levels can arguably be best understood as an oscillatory pattern created through a negative feedback mechanism (36–38). It can be hypothesized that the signaling of FSH inducing inhibin B and inhibin B suppressing FSH (i.e., FSH<->B) may not occur instantaneously and therefore result in oscillating levels. To test the hypothesis of an oscillatory relationship between FSH and B during the follicular phase, we employed functional data analysis (fda) which can be used to align subject data considered as functions of time (39, 40).

## 2 Materials and methods

### 2.1 Subjects, transvaginal sonography, and daily serum samples

Details on the four subjects and their four cycles, the periovulatory transvaginal sonography to establish the 24-h interval of the last day of the DF and first day of collapse, and the daily blood sampling have been previously published (41). The subjects and cycles here, S1, S2, S3, and S4, correspond respectively to 1Y1, 2Y1, 4Y1, and 6Y1 in publication (41). In summary: daily serum was obtained 830 a.m.–1130 a.m. starting from calendar day 1 (CD1) until the start of the next cycle during four ovulatory cycles from four subjects: 27-32 years of age, BMI of 18.6-26.2, and regular cycles of 25-28 days. A total of 105 serum samples were collected; only two samples, day −15 for cycle S1 and day −11 for cycle S3, were missed. The study was approved by the Institutional Review Board of TTUHSC #A23-4337, approval date 13 April 2023. Serum estradiol (E2), LH, and progesterone (P) levels for these cycles were previously reported (41) and obtained with the Abbott Architect ci4100 platform in the Panhandle Reproductive Research Lab, the clinical laboratory for the TTUHSC Amarillo clinics. Serum FSH levels were also obtained by batch and used in the present study. Transvaginal sonography every 10 a.m.–noon was started 7 days before the estimated day of ovulation and continued generally until 2 days of DF collapse. Transvaginal images and data storage were performed with a Phillips EPIQ 7 ultrasound machine in the Department of Obstetrics & Gynecology, TTUHSC Amarillo (41).

### 2.2 Indexing of the day-specific serum inhibin B and FSH levels to day 0, the day of the mid-cycle inhibin B peak

The event of ovulation—or rather the process of ovulation—was measured as the 24-h interval bounded by the last day of the DF and the next day of DF collapse; this interval was corroborated by serum P levels. Day 0 for publication (41) of cycles 1Y1, 2Y1, 4Y1, and 6Y1 (here S1, S2, S3, and S4, respectively) was the morning of DF collapse and rise in P to > 2 ng/ml. However, herein day 0 is the day of mid-cycle peak inhibin B. This was done to optimize the fda computations; also, published studies with mean inhibin B levels suggested that a mid-cycle inhibin B peak marks the day of ovulation (20–25). The mid-cycle inhibin B levels coincided with the day of DF collapse and serum P >2.0 ng/ml for cycles S1, S2, and S4. However, in cycle S3, the inhibin B peak occurred on the last day of the DF. Interestingly, the serum P level for cycle S3 on the last day of the DF was 2.4 ng/ml, suggesting that the DF was already in the process of rupture and luteinization and therefore the peak of inhibin B.

### 2.3 Inhibin B and inhibin A assays

Serum samples were kept at −80° until ready for batch assay in the laboratory of Harvinder Singh Gill, Department of Chemical Engineering, TTU, Lubbock. Inhibin B levels were determined with enzyme-linked immunoassay kits from Ansh Labs LLC, Webster, TX USA. These assays capture the corresponding β_B_ subunit and sandwiches this subunit specifically by detecting the α subunit.

### 2.4 Functional data analysis

Functional data analysis refers to a set of tools, including alignment, comparison, and statistical modeling developed to study complex, modern data objects that are represented as one-dimensional functions, shapes of curves and surfaces, and images (39, 40). In this study, the data, FSH, and inhibin B are functions of time, FSH(t) and B(t), where time is day of cycle indexed to day 0, the day of the ovulatory inhibin B peak. The FSH and inhibin B levels at least in the early follicular phase before selection of the DF were thought to be out-of-phase between cycles because of the differing number of antral follicles and differing stage of development (1–5). With the aim of extracting meaningful summary statistics—i.e., summary curves for FSH(t) and B(t)—one of the biggest challenges is the alignment step (42). The alignment step of fda with warping functions reduces the lateral displacements in curve features due to phase variations. To accomplish these computations, we used the dynamic time warping algorithm recently developed by Srivastava and Klassen (39) and implemented in the R package “fdasrvf” as elastic fda (43). Once the FSH and inhibin B curves were aligned, since measurements were at the same time points, cross-sectional mean day-specific and standardized cross-sectional mean day-specific curves were generated.

Standardized cross-sectional curves were as follows: (FSH or inhibin B level for time point t - Mean level of FSH or inhibin B for the cycle)/standard deviation). The standardized cross-sectional mean day-specific curves (levels) are herein referred to as “standardized mean day-specific” curves (levels) for FSH and inhibin B.

From the 24-h blood sampling, the computed standardized mean day-specific curves for FSH and inhibin B appeared to be out-of-phase by 1 day. A positive effect of FSH on inhibin B levels where FSH lagged by 1 day was tested by translating the FSH curve forward by +1 day and correlating with the inhibin B curve (i.e., FSH(t+1) vs. B). Conversely, a suppressive effect of inhibin B on FSH levels where inhibin B lagged by 1 day was tested by translating the inhibin B curve forward by +1 day and correlating it with the FSH curve (i.e., B(t+1) vs. FSH). The correlations between these curves were calculated using the classical Pearson, Kendall, and Spearman correlation coefficients (44).

## 3 Results

### 3.1 Day-specific serum FSH and inhibin B levels for four individual cycles

**Figure 1:**
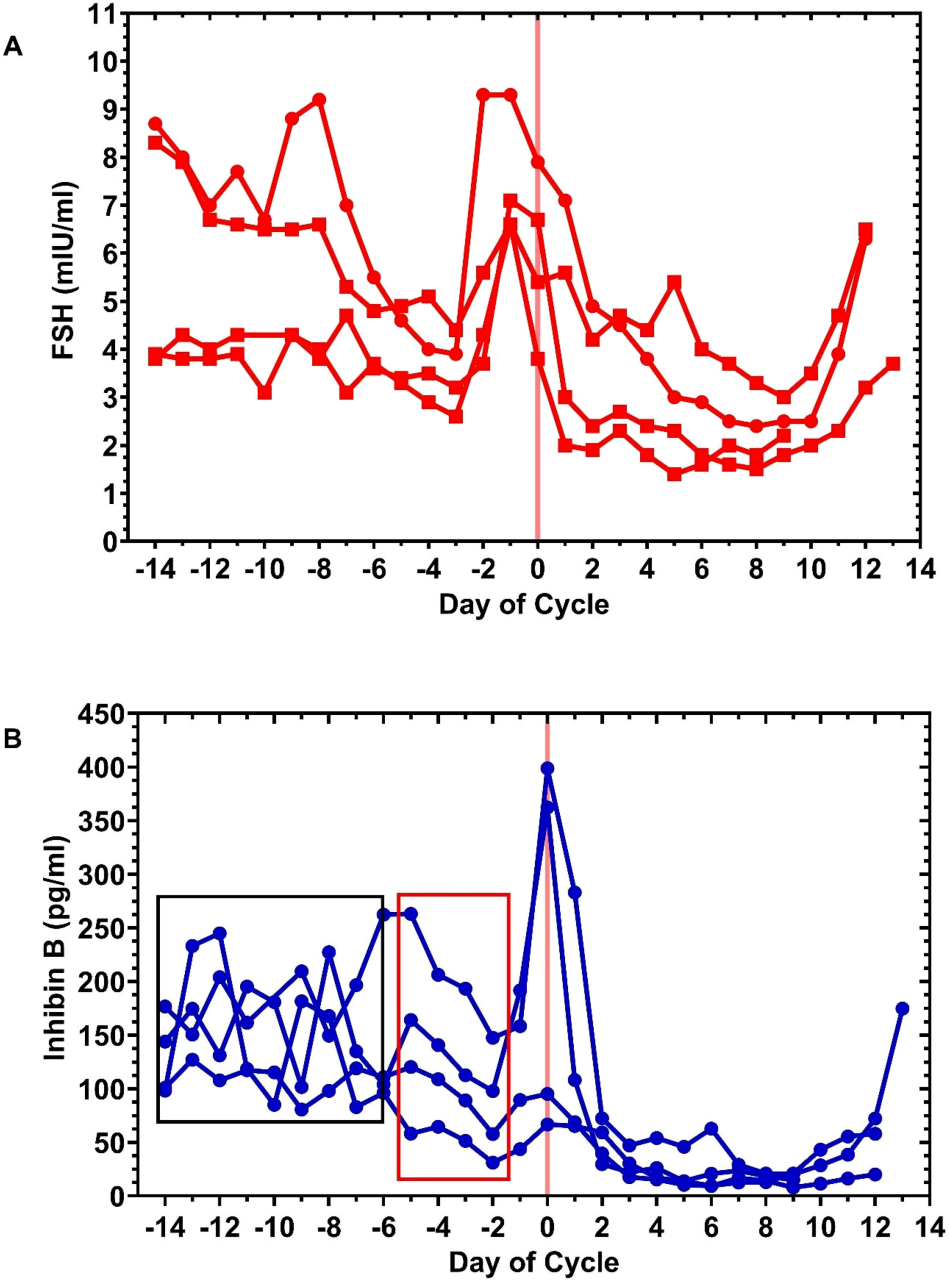
Day-specific serum FSH and inhibin B levels during ovulatory cycles, S1, S2, S3, and S4, from four subjects. These levels are shown in [**(A)**, top] and [**(B)**, bottom], respectively. The four cycles are indexed to day 0 (pink vertical line), the ovulatory peak of inhibin B, which occurred in the 24-h interval bounded by the last day of the DF and the following day of sonographic DF collapse. For cycles S1, S2, and S4, the ovulatory peak of inhibin B occurred on the day of DF collapse with serum P levels: 1.3 ng/ml (4.13 nmol/L), 0.7 ng/ml (2.23 nmol/L), and 1.8 ng/ml (5.72 nmol/L), respectively, and DF sizes 21.8, 28.0, and 26.9 mm, respectively. For cycle S3, the ovulatory peak of inhibin B occurred on the last day of the DF (size 22.4mm), but the P level was 2.4 ng/ml (7.63 nmol/L), indicating that the DF was advanced toward luteinization and close to or in the process of rupture. Day 0 peak inhibin B levels for S1, S2, S3, and S4 were 362.34, 66.61, 398.89, and 95.23 pg/ml, respectively. An oscillatory pattern for inhibin B was notable in the early follicular phase for these cycles variably ending at day −6 (black box) with a fairly linear decline in levels within the anticipated fertile window (red box) with a minimum at day −2.

Four subjects provided daily blood samples for an ovulatory cycle, cycles S1, S2, S3, and S4. Serum levels of FSH and inhibin B are shown in **Figure 1** and indexed to the peri-ovulatory peak of inhibin B. The published inhibin B signature, a mean of many cycles, has shown this peak at the time of presumptive ovulation (20–25). In order to optimize the fda of these levels, the levels were indexed to this ovulatory inhibin B peak. The ovulatory event—release of an ovum—occurs in the 24-h interval between the last day of the DF and the next day of sonographic collapse of the DF. For cycles S1, S2, and S4, the day of inhibin B occurred the day of the DF collapse and in S3 the inhibin peak was on the last day of the DF. Interestingly, for cycle S3, the serum progesterone level measured the last day of the DF was already significantly elevated at 2.4 ng/ml, perhaps suggesting eminent release of the ovum.

The inhibin B ovulatory peak levels ranged 66.6–398.8 pg/ml. In all the cycles, the pre-ovulatory FSH peak, which ranged 6.6–9.3 mIU/ml, occurred 1 day before the inhibin B ovulatory peak.

### 3.2 Functional data analysis of day-specific inhibin B and FSH levels

**Figure 2:**
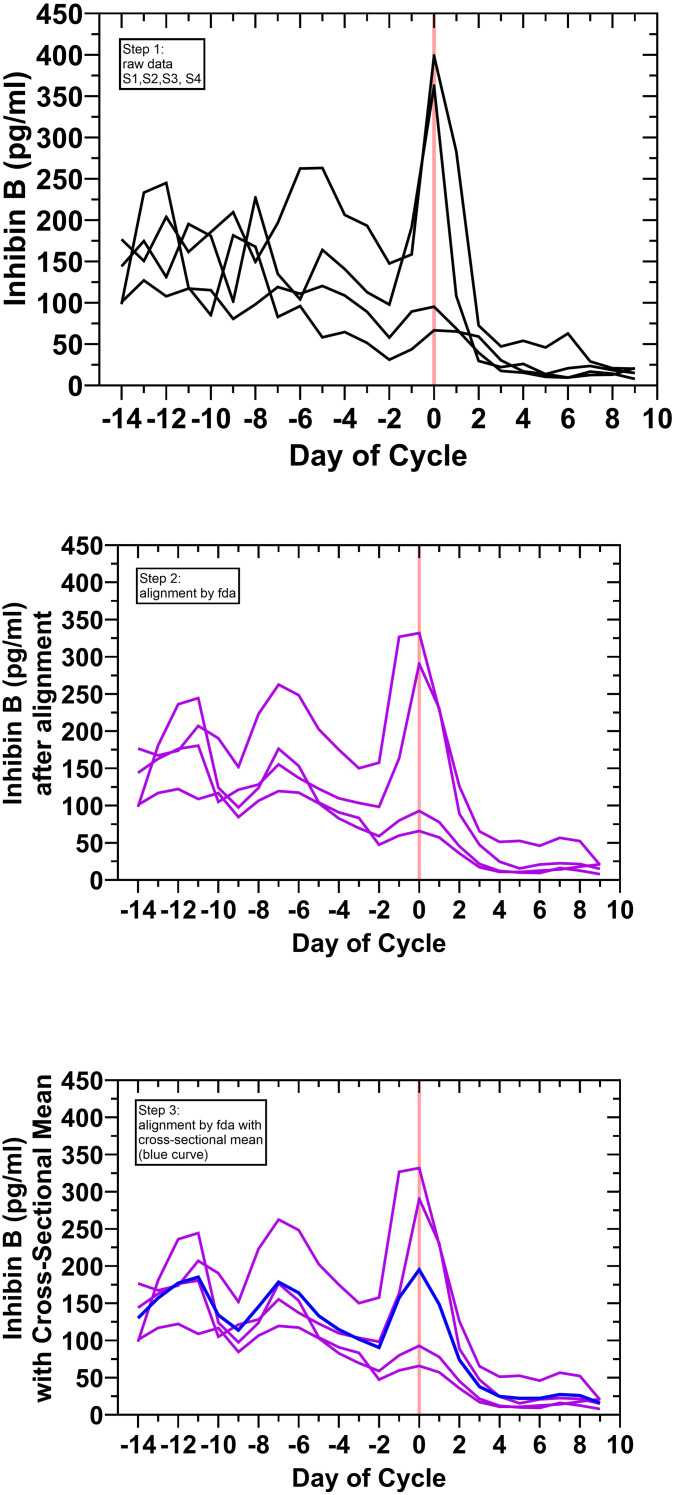
Illustration of the application of functional data analysis (fda) using the inhibin B levels for cycles S1, S2, S3, and S4. As an example of the fda process: step 1 (top panel and **Figure 1**)—the raw inhibin B levels as a function of time of cycle were first obtained; step 2 (middle panel)—these curves were then aligned using the ovulatory inhibin B peak as day 0 (pink vertical line); step 3 (bottom panel)—the mean of the aligned curves generated the cross-sectional mean day-specific inhibin B curve (blue). The alignment step of fda (called “registration”) seeks to synchronize curves measured over time that can plausibly be viewed as being out-of-phase due to random variability or noise. This is accomplished through individual time deformations (warping) of each curve, and subsequent optimization of an objective function (the Fisher-Rao metric) which ultimately yields an “optimal” synchronization for the entire group of curves. This can be heuristically understood as allowing the curves to deform until a maximal “correlation” among the group is achieved (39).

A feature of the individual cycles was the apparent oscillatory pattern of inhibin B in the early follicular phase which ended at mid-cycle, the predicted timing of DF appearance; this is indicated in **Figure 1**. There was also a suggestion of this, albeit to a lesser degree, with inspection of the early follicular phase FSH levels. Rather than random scatter, the possibility of out-of-phase inhibin B and FSH levels between cycles due to variable antral follicular waves was considered, and therefore, fda was employed to investigate this hypothesis. An illustration of the fda method is shown in **Figure 2** for the day-specific inhibin B levels. It was hypothesized that an inhibin B and FSH interaction might be obscured by out-of-phase factors between cycles, but a relationship could be potentially visualized by fda. Functional data analysis uses warping functions which capture the phase variation in the original curves and optimizes the fit of the aligned curves (39, 40, 42–44). The final results for analysis derived from this methodology were as follows: a) cross-sectional mean day-specific levels for inhibin B and FSH and b) standardized (normalized) cross-sectional mean day-specific FSH and inhibin B levels, referred herein to as standardized mean curves or standardized mean day-specific curves for brevity.

**Figure 3:**
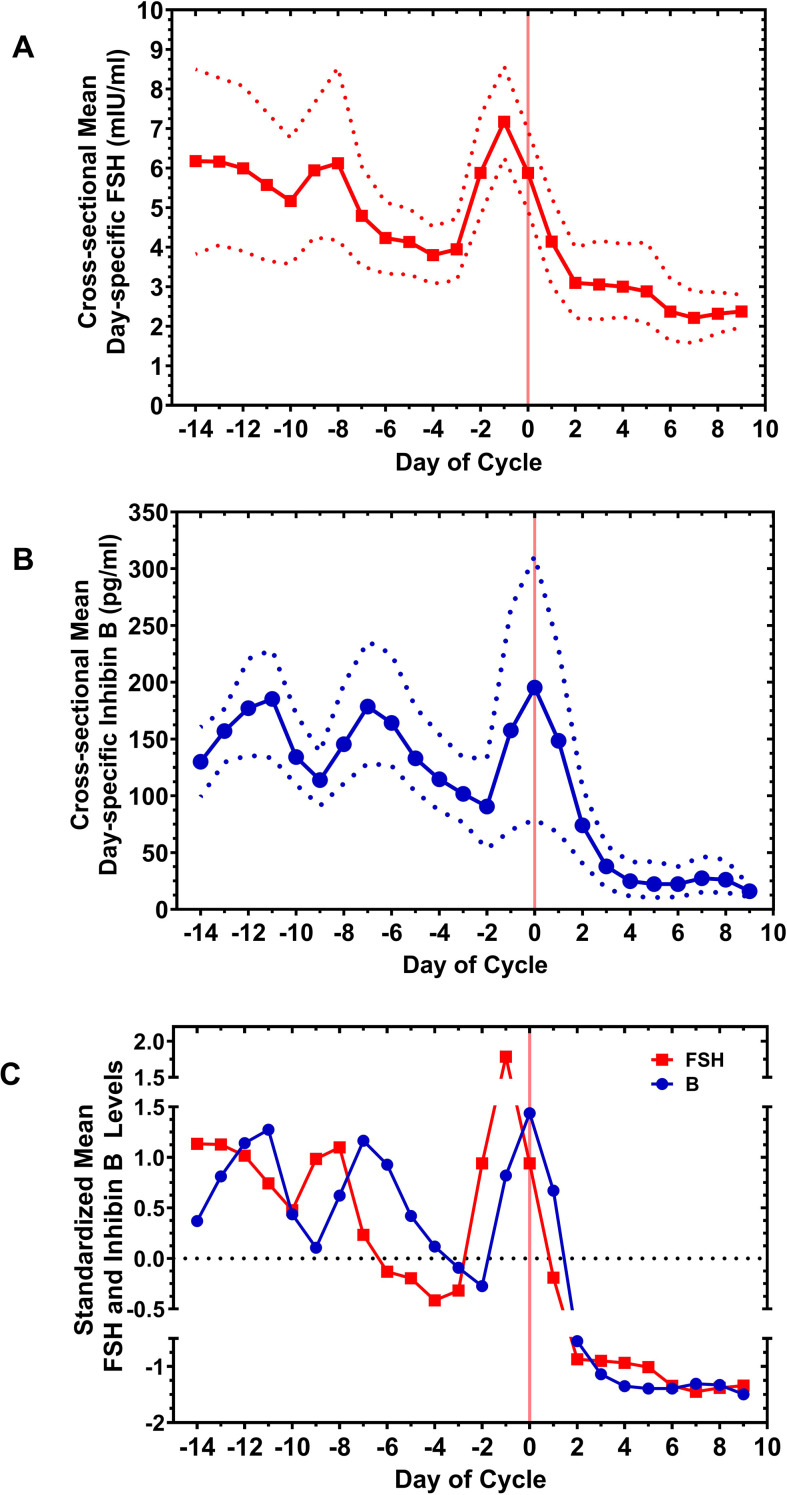
Functional data analysis (fda) of the FSH and inhibin B levels for cycles S1, S2, S3, and S4. The processing of the FSH and B curves from **Figure 1** by fda is detailed in the Materials and Methods and illustrated for the inhibin B levels in **Figure 2**. With this methodology, cross-sectional mean day-specific FSH and inhibin B levels (solid lines) with 95% confidence intervals (dotted lines) were determined [**(A)**, top and **(B)**, middle, respectively). For comparative purposes, standardized cross-sectional mean (“standardized mean”) day-specific FSH and inhibin B levels (curves) were determined and displayed together [**(C)**, bottom]. An oscillatory pattern in the early follicular phase was further explored by correlating the FSH and inhibin B curves in **Figure 4**.

The signature curves for the cross-sectional mean day-specific inhibin B and FSH levels and the corresponding standardized mean day-specific FSH and inhibin B levels are shown in **Figures 3A–C** respectively. The dotted lines in **Figures 3A, B** correspond to 95% confidence intervals for the cross-sectional means (solid lines) which comprise the signature curves. These confidence intervals were computed via the “nonparametric bootstrap” (percentile method), a methodology appropriate for small sample situations, thus providing a measure of uncertainty quantification given that each mean is based on only four values (45).

The relationship of the standardized mean inhibin B and FSH curves plotted together suggested a periodic function that changed after day −8 (**Figure 3C**). The interval from approximately day −7 to day 0, the time anticipated for DF development and ultimate ovulation, displayed distinctly different inhibin B and FSH functions compared with the early follicular phase, day −14 to day −8. After day 0, the FSH and inhibin B levels were low, consistent with the published average levels for multiple cycles (20–25).

### 3.3 Functional data analysis shows a changing oscillatory interaction between FSH and inhibin B during the follicular phase

**Figure 4:**
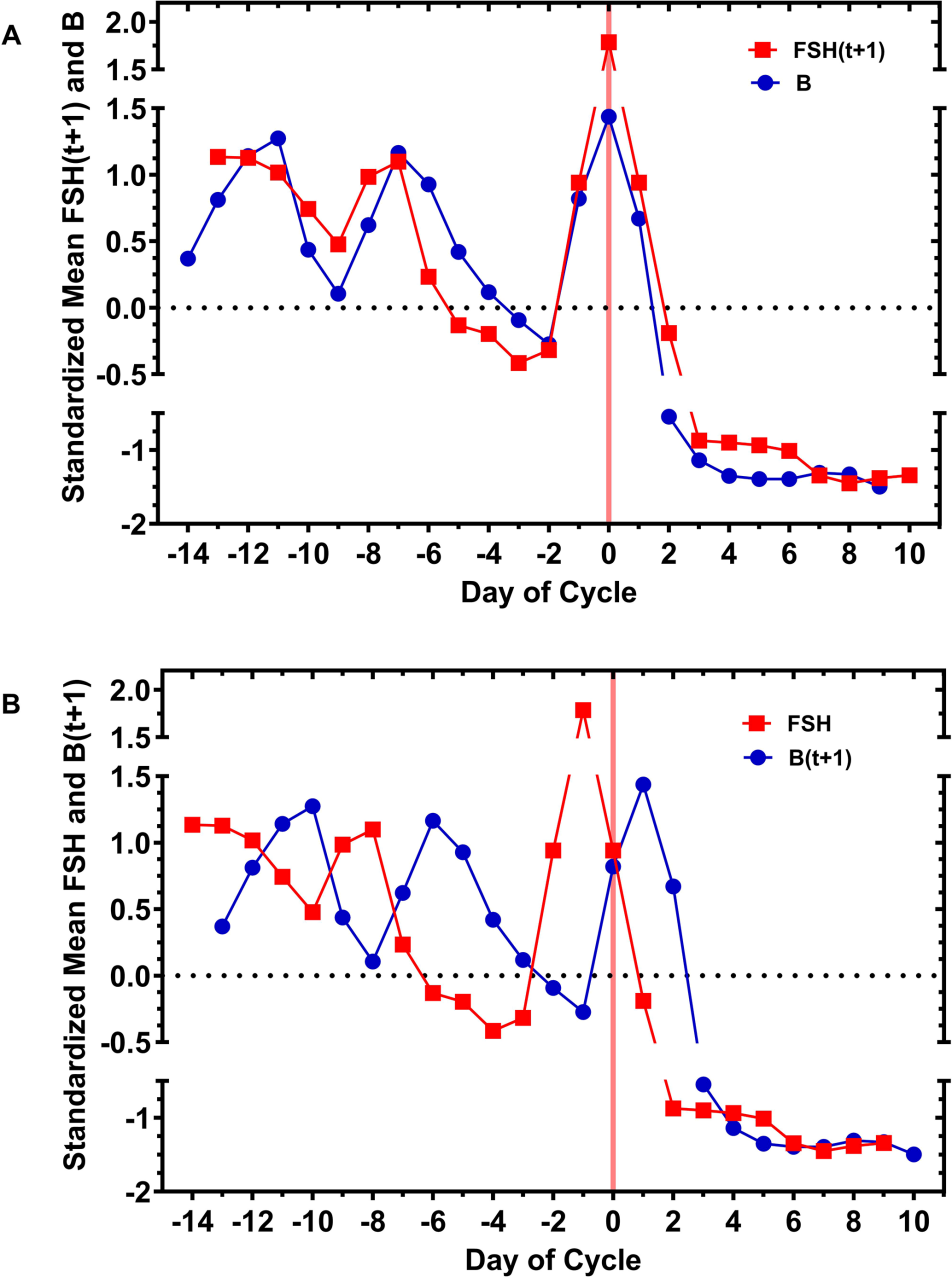
Translation of the standardized mean FSH curve by +1 day, FSH(t+1), vs. the standardized mean inhibin B curve and translation of the standardized mean inhibin B curve by +1 day, B(t+1), vs. the standardized mean FSH curve [**(A)**, top and **(B)**, bottom, respectively]. Positive regulation of inhibin B by FSH was examined by moving the FSH curve forward by 1 day of the cycle (i.e., FSH(t+1)) in relation to the inhibin B curve. There was considerable overlap in the curves of the standardized mean levels during all phases of the cycle, suggesting that FSH upregulates inhibin B throughout the ovulatory cycle and that this FSH effect was demonstrable at 1-day intervals. Conversely, the negative regulation of FSH by inhibin B was examined by moving the inhibin B curve forward by 1 day of the cycle (i.e., B(t+1)). With the B(t+1) translation in relation to FSH, an oscillatory positive-negative pattern during the early follicular phase was manifested, which qualitatively changed after approximately day −8. Day 0 (pink vertical line) is the ovulatory inhibin B peak, which occurred on the last day of the DF (1/4 cycles) or the day of DF collapse (3/4 cycles).

The possibility of a positive–negative feedback loop between FSH and inhibin B, FSH<->B, was further investigated by translating the FSH and inhibin B curves on the same plots (**Figures 4A, B**). The top plot (**Figure 4A**) shows the standardized mean day-specific FSH curve advanced by 1 day (FSH(t+1)) compared with the standardized mean day-specific inhibin B curve, and conversely, the bottom plot (**Figure 4B**) shows the standardized mean day-specific inhibin B curve (B(t+1)) advanced by 1 day (B(t+1)) compared with the FSH curve. Given that serum measurements were on a 24-h basis, these translations examined the hypothesis that a positive, up-regulation effect of FSH on inhibin B could be seen after 1 day, and conversely, the negative, suppressive effect of inhibin B on FSH could be seen after 1 day, resulting in an oscillatory pattern. Surprisingly, after this overlay, the FSH(t+1) curve tracked the inhibin B curve throughout the entire cycle suggesting upregulation by FSH. In contrast, an oscillatory pattern between inhibin B and FSH which changed at approximately day −8 to day −7 suggested downregulation by inhibin B. Pearson, Kendall, and Spearman coefficients for the entire cycle, day −13 to day +9, for the curves FSH(t+1) vs. inhibin B were 0.95 (P<0.001), 0.77 (<0.001), and 0.92 (<0.001), respectively. A strong negative correlation for the curves inhibin B(t+1) vs. FSH was found for the oscillatory pattern between days −13 and −8; the corresponding Pearson, Kendall, and Spearman coefficients with P values were −0.86 (P 0.02), (−0.73, P 0.05), and (−0.89, P 0.03), respectively. Taken together, the data suggest a potential FSH<->B mechanism, a positive–negative feedback loop, that qualitatively changes near the time of DF selection.

## 4 Discussion

### 4.1 FSH<->B from an exploratory study with fda: a proposed positive–negative oscillatory feedback loop between FSH and inhibin which changes during the follicular phase

The interaction of FSH and inhibin B during the follicular phase leading up to ovulation has been analyzed through many studies, but usually on one level, that of the linear relationship between mean FSH and mean inhibin B under variable conditions. These studies have convincingly shown that FSH upregulates inhibin B at least in the follicular phase (32–35). The important role of inhibin B as a negative regulator of FSH has also been proposed (3, 31).

What has not been so well studied is an oscillatory interaction between FSH and inhibin B. A single prior publication studied five normal women on a single day, CD 5, by sampling blood every 10 min for 6 h and measuring serum FSH and inhibin B levels (46); time series analysis demonstrated the presence of 60–70 min periodicity of inhibin B levels and, similarly, a pulsatile pattern of FSH levels every 60 min. In the present study, four individual ovulatory cycles were investigated where the serum FSH and inhibin B levels were indexed to the day the inhibin B peak. This inhibin B peak was the first day of DF collapse in 3 of 4 cycles and the last day of the DF in 1 cycle; this interval encompasses the ovulation event. The apparent saw tooth scatter of the early follicular phase inhibin B and FSH levels (**Figure 1**) was conjectured to be due to out-of-phase follicular waves, and the approach taken to solve FSH and inhibin as a function of time was fda. With this method of analysis, the standardized mean day-specific FSH and inhibin B levels suggested oscillation between FSH and inhibin B that changed after day −8, the timing of DF selection. Our results obtained from 24-h sampling do not preclude the possibility of a higher frequency of oscillation of FSH<->B, that is, a finer structure to the oscillator function, during shorter time intervals.

What causes the FSH<->B oscillatory pattern to change at midcycle before ovulation can only be speculated. Perhaps rising levels of E2 and inhibin A which occur at this time predominate over inhibin B in terms of FSH suppression. The suppressive effect of E2 and/or inhibin A may explain the lesser concordance of the standardized mean FSH and inhibin B levels seen between day −6 to day −3 (**Figure 4A**), although the slope of the curves is similar suggesting some continued positive FSH effect on inhibin B levels.

One aspect of this study is demonstration of the usefulness of fda in analyzing data with individual cycles. In considering qualitative mathematical modeling of endocrine systems, an important general point is that single subjects, here single cycles, are important since secretion patterns between subjects/cycles can be out-of-phase (37, 47). Functional data analysis may be a useful method to discern meaningful relationships when secretion patterns between subjects/data sets, as the case with FSH and inhibin B, are out of phase (39, 40, 42, 43). The concept here is that there is a collection of related antral follicles undergoing progression as a wave under the influence of FSH (1, 2). Inhibin B is secreted from the granulosa cells of the developing follicles (3). There are several variables that contribute to a feedback oscillatory function. First, there is a variable number of developing follicles, ~4–14 (2). Second, they are in a variable state of development, and the level of inhibin B secretion is known to correlate with size (3, 25–29). Third, individual antral follicles probably see different levels of FSH, that is, a variable time delay factor, because of variable blood flow (48, 49). The resultant of these differences would be out-of-phase FSH and inhibin B levels between different subjects on a specific day during the early follicular phase before DF appearance. Functional data analysis of the cumulative data from the four cycles enabled appropriate phasing and computation of standardized mean curves.

FSH and inhibin B levels were determined by different assay systems, and yet there was a considerable correlation between the FSH and inhibin B curves determined by fda contributing to the authenticity of the results described here. However, a limitation of this study is that only four cycles were analyzed. Further work could reveal fine structure to the FSH and inhibin B curves and other regulatory features.

Interestingly, a prior publication has noted the problem with combining fluctuating hormone data (E3G (estrone-3-glucuronide) and LH) from different patients on the same graph because “shifts of the phases of oscillations between different patients cancel each other” (50). Furthermore, the authors suggest that oscillations between gonadotropins and E2 may be a factor in “survival of the fittest follicle”. This needs to be further explored along with the apparent oscillatory behavior between FSH and inhibin B. In addition, the set point of the hypothalamic–pituitary axis can be altered as seen in the thyroid function of Klinefelter syndrome as a function of age, and this would affect oscillations (51).

#### 4.2.1 Oscillatory behavior as a potential mechanism for regulating ovarian antral follicular wave dynamics and DF selection

Oscillatory functions—that is, periodic hormone levels with time—may be the preferred mode of transmitting information in physiological systems (36, 37, 47, 52). How oscillatory FSH<->B could regulate a wave of individual antral follicles and end in a DF is beyond the scope of this work. Hypothetically, variable oscillating FSH levels in amplitude and frequency seen by individual follicles in waves could be a factor in DF selection, although a mechanism for this is presently unknown. FSH may have important effects on extra-gonadal tissues (prostate, adipose, cardiac, bone, immune cells), and thus oscillatory behavior could be important in signaling in these non-canonical tissues (53).

A limitation of this study is that follicle size and number were not subjected to fda analysis with corresponding FSH and inhibin B levels. A data matrix of developing antral follicle size and number determined by sequential sonograms with FSH and inhibin B levels as a function of time during the follicular phase might provide further insight into the role of oscillatory regulation. Further limitations of this pilot study are that it included only four cycles and did not study variability of the FSH<->B within the same woman. In addition, blood sampling occurred at only one time interval in the morning and other potential variables such as stress and sleep were not addressed.

In summary, functional data analysis of four individual cycles suggested that FSH upregulates inhibin B levels throughout the ovulatory cycle, albeit other factors affect inhibin B levels as well. Furthermore, during the early follicular phase before selection of the DF, an oscillatory relationship between FSH and inhibin B was seen. It is speculated that a property of an oscillatory positive–negative feedback loop, FSH<->B, may be important in the development of ovarian follicular waves leading to a DF.

## Data Availability

The original contributions presented in the study are included in the article/supplementary material. Further inquiries can be directed to the corresponding author.
